# Artificial Intelligence in Skin Cancer Diagnostics: The Patients' Perspective

**DOI:** 10.3389/fmed.2020.00233

**Published:** 2020-06-02

**Authors:** Tanja B. Jutzi, Eva I. Krieghoff-Henning, Tim Holland-Letz, Jochen Sven Utikal, Axel Hauschild, Dirk Schadendorf, Wiebke Sondermann, Stefan Fröhling, Achim Hekler, Max Schmitt, Roman C. Maron, Titus J. Brinker

**Affiliations:** ^1^Division of Translational Oncology, National Center for Tumor Diseases (NCT), German Cancer Research Center (DKFZ), Heidelberg, Germany; ^2^Division of Biostatistics, German Cancer Research Center, Heidelberg, Germany; ^3^Department of Dermatology, Heidelberg University, Mannheim, Germany; ^4^Skin Cancer Unit, German Cancer Research Center, Heidelberg, Germany; ^5^Department of Dermatology, University Hospital Schleswig-Holstein, Kiel, Germany; ^6^Department of Dermatology, University Hospital Essen, Essen, Germany

**Keywords:** artificial intelligence, patients view, melanoma, diagnostics, acceptance, skin cancer, online survey

## Abstract

**Background:** Artificial intelligence (AI) has shown promise in numerous experimental studies, particularly in skin cancer diagnostics. Translation of these findings into the clinic is the logical next step. This translation can only be successful if patients' concerns and questions are addressed suitably. We therefore conducted a survey to evaluate the patients' view of artificial intelligence in melanoma diagnostics in Germany, with a particular focus on patients with a history of melanoma.

**Participants and Methods:** A web-based questionnaire was designed using LimeSurvey, sent by e-mail to university hospitals and melanoma support groups and advertised on social media. The anonymous questionnaire evaluated patients' expectations and concerns toward artificial intelligence in general as well as their attitudes toward different application scenarios. Descriptive analysis was performed with expression of categorical variables as percentages and 95% confidence intervals. Statistical tests were performed to investigate associations between sociodemographic data and selected items of the questionnaire.

**Results:** 298 individuals (154 with a melanoma diagnosis, 143 without) responded to the questionnaire. About 94% [95% CI = 0.91–0.97] of respondents supported the use of artificial intelligence in medical approaches. 88% [95% CI = 0.85–0.92] would even make their own health data anonymously available for the further development of AI-based applications in medicine. Only 41% [95% CI = 0.35–0.46] of respondents were amenable to the use of artificial intelligence as stand-alone system, 94% [95% CI = 0.92–0.97] to its use as assistance system for physicians. In sub-group analyses, only minor differences were detectable. Respondents with a previous history of melanoma were more amenable to the use of AI applications for early detection even at home. They would prefer an application scenario where physician and AI classify the lesions independently. With respect to AI-based applications in medicine, patients were concerned about insufficient data protection, impersonality and susceptibility to errors, but expected faster, more precise and unbiased diagnostics, less diagnostic errors and support for physicians.

**Conclusions:** The vast majority of participants exhibited a positive attitude toward the use of artificial intelligence in melanoma diagnostics, especially as an assistance system.

## Introduction

Systems based on artificial intelligence, particularly on deep learning, are entering the medical field at an impressive pace. Layered mathematical models process large datasets to perform a specific task, for example automatic pattern recognition (1). Image analysis is one of the areas in which the fastest progress is currently achieved (2), for instance in the detection and quantification of lung nodules on radiological images (3), image-based detection of potential strokes (4) or breast cancer screening (5).

In the field of dermatology, Artificial intelligence-based tools are being developed to evaluate the severity of psoriasis (6) or to distinguish between onychomycosis and healthy nails (7, 8). In experimental settings, the sensitivity and specificity of AI-based algorithms in discriminating melanomas from nevi were similar to or better than those of dermatologists (9–11). Since the detection of melanomas at an early stage greatly improves prognosis and the distinction between melanomas and harmless lesions is often not trivial, AI-based classification systems might yield enormous benefits for patients with suspicious skin lesions.

However, there is still some controversy surrounding the use of AI for diagnostics in “real life” clinical settings. Concerns include the possibility of biases, the lack of transparency and explainability, scalability, data integration and interoperability, reliability, safety, privacy and ethics of aggregated digital data (12, 13).

The success of AI applications in clinical diagnostics depends on acceptance by both physicians and patients, which is driven by their understanding of the potential benefits and harms of AI. Patients' needs regarding medicinal aspects, but also data security, may differ from experts' expectations (14, 15). Therefore, physicians and patients have to be involved in the AI research agenda at an early stage to ensure that their needs are addressed adequately during the development of such applications.

A recent study in Korea has shown that in general, Korean medical doctors have a positive attitude toward AI in medicine (16). Similar results were obtained in a large international survey among dermatologists, indicating that AI is well-accepted in the dermatology field: The majority of participants agreed or strongly agreed that AI will improve dermatology and felt that AI should be a part of medical training (17). A survey on computer-assisted diagnosis of melanomas with dermatofluoroscopy also showed a high acceptance of that technology among patients (18). A Swedish survey among participants in a breast cancer screening program about AI-based automated mammogram analysis showed a high level of confidence in computer-aided decision making (19). A study on skin cancer-related apps revealed that around 43% of the participants thought that these apps might be able to supplement the professional skin examination. However, almost all patients had never used skin cancer-related apps or could not remember (20).

Here, we report the results of a survey-based cross-sectional study designed to investigate the hopes and fears of patients with and without a history of melanoma toward the use of AI in skin lesion diagnostics, especially with respect to the classification of melanoma.

## Methods

### Survey

We conducted an anonymous online survey, using the open source software LimeSurvey (Version 3.17.3.). The questionnaire was designed de novo as we were not aware of any validated survey tools for the objective of our study. After conducting a literature review we designed a questionnaire addressing the main topics and follow-up questions. These questionnaires were sent to dermato-oncological experts for annotation. After revisions, we tested the survey on five volunteers who had no professional background in artificial intelligence to review the comprehensibility and consistency, and revised it further. In addition to sociodemographic questions (age, sex, previous history of melanoma diagnosis, educational level), the questionnaire contained various items dealing with expectations and concerns regarding the use of artificial intelligence in melanoma diagnostics in general and in different application scenarios. For most questions, there were multiple choice response options, few questions were designed to fill in free text. This strategy ensures high interpretability, comprehensibility, and consistency in representation.

The survey was conducted from November 2019 to January 2020. We used an online survey to avoid the influence of study directors and to ensure anonymity (21).

As we wanted to compare the attitudes and opinions of patients with and without a history of melanoma the questionnaire was distributed via email to our dermatological university hospital cooperation partners and melanoma support groups with the request for further dissemination. In addition, it was advertised on social media. We did not do any formal hypothesis testing in the paper, and therefore also did not do a power calculation in the original sense. However, the sample size of at least 250 was planned in order to obtain standard errors of +/−2.5% or, equivalently, confidence intervals of about +/−5% for our rate estimates (see section “Data Analysis”). We closed the online LimeSurvey when we had surpassed that number of full-answer sets.

Participation was voluntary and anonymity was ensured by design. This might lead to an increased motivation to answer the questions thoroughly and truthfully and thereby enhance the intrinsic data quality. Every participant agreed with LimeSurvey's privacy policy. All data were stored at DKFZ. Participants were informed that the results of the survey would be used for scientific publication. All questions were optional and were available in German only. The survey received approval by the board of data protection at the German Cancer Research Center (DKFZ). For most questions, there were 5 response options: “agree entirely,” “rather agree,” “unsure,” “rather disagree,” and “disagree entirely.” Questions regarding concerns and expectations were designed to fill in free text (see questionnaire in the Appendix in German).

### Data Analysis

Three hundred and sixty one data sets were generated. Sixty three data sets were excluded in the analysis as they were only filled in to an extremely limited extent (less than half of the questions answered). Two hundred and ninety eight data sets were included in the data analysis: 292 of them were completed up to the end, 6 were completed except for the last page (last two questions). All questions were optional, 140 participants answered every question.

Descriptive analysis was performed with interpretation of categorical variables as percentages. For simplified descriptive statistics, the categories “rather agree” and “agree entirely” were summarized as agreement while “rather disagree” and “disagree entirely” were summarized as disagreement. The category “unsure” was reported separately. For statistical analyses on associations between sociodemographic data and selected items of the questionnaire, the original categories were preserved, in order to obtain a better differentiation to best reflect the comparison between the subgroups. We pre-specified sub-group analyses on age, education, gender, and participants with and without a history on melanoma and collected these personal data in the anonymous survey. Subgroup analyses were conducted for each question. In the results section, we report only those in detail where there was a significant difference.

Ninety five percentage confidence intervals were computed for the main results using the normal distribution approximation.

The margin of error (standard error) for percentage rates estimated from *n* = 298 participants is +/−2.5 percentage points at rates of about 80%.

Statistical analysis was done using Microsoft Excel and SigmaPlot. Comparison of the distribution of responses across sub-groups was performed using the Wilcoxon signed rank or the chi^2^ test. In data analyses with chi^2^ tests, participants with no answer to the relevant question were not included. A *p* < *0.05* was regarded as statistically significant.

## Results

### Baseline Characteristics of the Study Population

Over a 10 week period, a total of 298 individuals responded to the questionnaire. About half of the respondents reported a previous melanoma diagnosis, almost three quarters were female and almost two thirds graduated from high school with German “Abitur” or had a university degree (Table 1). This is not representative for the situation in the general population.

**Table 1:** Baseline characteristics of the study population.

		**% (*n* = 298)**
**Gender**		
Female	218	73.2
Male	73	24.5
Diverse/Not reported	7	2.3
**Previous diagnosis of melanoma**		
Yes	154	51.7
No	143	48
Not reported	1	0.3
**Age**		
≤ 30	41	13.8
31–45	102	34.2
46–60	123	41.3
61–75	27	9.1
>75	4	1.3
Not reported	1	0.3
**Education**		
Graduated from general secondary school (“Hauptschulabschluss”)	19	6.4
Graduated from intermediate secondary school (“Realschulabschluss”)	77	25.8
Higher school certificate qualifying for university admission (“Abitur”)	77	25.8
University degree	121	40.6
Not reported	4	1.3

### General Awareness of and Attitude Toward the Use of AI

**Figure 1:**
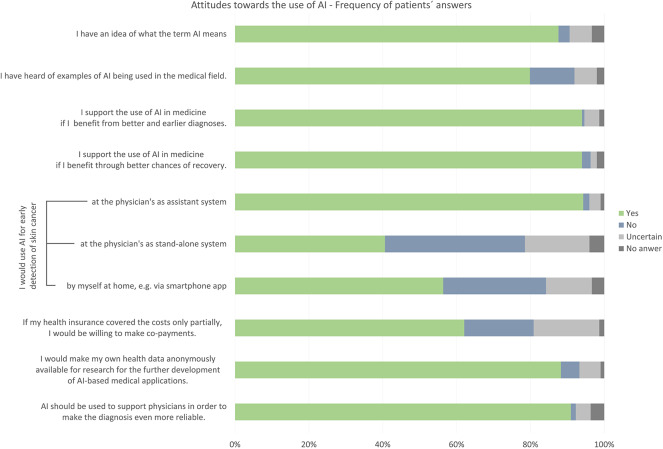
General attitude toward the use of Artificial Intelligence (AI) in the medical field. Bar chart shows distribution of participants' answers regarding their awareness of and attitude toward AI in medical use, provided that the AI-based system can distinguish well between images of harmless moles and melanoma.

Of all respondents, 88% had already heard of artificial intelligence and had a general idea on what AI signifies (3% did not, 6% were uncertain, 3% did not answer the question). Less respondents (80%) had explicitly heard of examples of AI in use in medical applications (12% did not, 6% were uncertain, 2% did not answer this question). The vast majority of the respondents (94, 95% CI = 0.91−0.97) stated that they would support the use of AI in medicine. Only 1.3% explicitly did not agree with the statement that AI should be used to support physicians to make skin cancer diagnostics even more reliable (91% agreement, 4% uncertain, 3.7% no answer). Eighty eight percent [95% CI = 0.85–0.92] claimed that they would even make their own health data anonymously available for the further development of AI-based medical applications (5% disagreement, 6% uncertain, 1% no answer). If an AI-based system was able to distinguish well between images of nevi and melanoma, a vast majority of respondents would be willing to use AI for early detection of skin cancer in some way. In particular, 94% [95% CI = 0.92–0.97] would endorse the use of AI as assistance system at the physician's (2% disagreement, 3% uncertain, 1% no answer). At least 41% [95% CI = 0.35–0.46] would also consider its use at the physician's as a stand-alone system (38% disagreement, 17% uncertain, 4% no answer) and 56% stated that they might use AI-based applications even at home, e.g., as a diagnostic smartphone app (28% disagreement, 13% unsure, 3% no answer, Figure 1).

### Confidence in Artificial Intelligence vs. Medical Doctors

**Figure 2:**
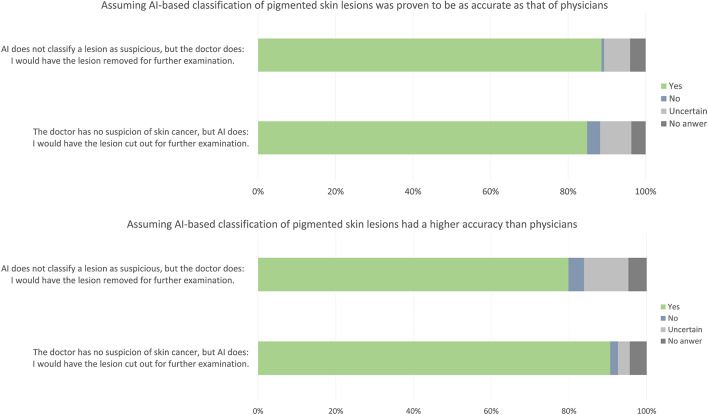
Confidence in AI. Bar chart shows the frequency of participants' answers.

If the AI-based classification of pigmented skin lesions was proven to be equally accurate as that of physicians, respondents had slightly more confidence in the physician's decisions than in those of the AI algorithm. If the physician considered a tissue biopsy or an excision necessary, but the algorithm did not, 89% of respondents would agree to the operation (the disagreement was <1%, around 7% were unsure, 4% did not answer the question), whereas 85% would do so in the reverse case (3% disagreement, 8% unsure, 4% no answer). However, if it was proven that AI-based classification systems were more accurate than physicians, most respondents would rely on the algorithm rather than on the physician: 91% of respondents would agree to a biopsy/excision if the algorithm rated this as necessary and the physician did not (2% disagreement, 3% uncertain, 4% no answer), whereas only 80% would do so in the reverse case (4% disagreement, 11% uncertain, 5% no answer). Overall, most participants responded that they would have the lesion biopsied or excised in any case (> 80%, Figure 2).

### Concerns Regarding AI

Questions regarding concerns and expectations were designed to fill in free text.

The concerns most frequently voiced in this survey were related to data protection, impersonality and susceptibility to errors.

Participants feared that their data might not be anonymized, and hence might be misused to their disadvantage by health and other insurance companies or employers. Also, there was a concern that personal data might be published by hackers.

Another common concern regarding the use of AI was a diminished physician-patient relationship—consultations could become more sterile, with no personal conversation with the physician and less time for questions. Respondents were also worried that physicians might be tempted to rely on the AI-based algorithm so much that they would lose their own expertise and diagnostic skills. This would reduce their ability to classify lesions without the assistance system as well as their ability to notice obvious mistakes or malfunctioning of the algorithm itself, which could occur due to various technical problems or even deliberate manipulation by hackers.

Further perceived problems that were mentioned were the non-traceability of the decision algorithms and the missing transparency of the applied systems. In addition, the risk of unequal opportunities due to potential high costs not covered by standard health insurance was pointed out.

### Hopes and Expectations Regarding Artificial Intelligence

Participants expected that by integrating AI algorithms into skin cancer diagnostics, waiting time due to the requirement for tissue analysis by the pathologist might be reduced. Many participants also hoped that AI would lead to earlier detection of skin cancers, maybe even at the precursor stage, thereby decreasing the required therapeutic intensity for those lesions. Moreover, they stated that the use of AI might lead to more reliable and less subjective diagnoses, which might lead to fewer unnecessary biopsies and less overlooked malignant lesions. As AI offers the possibility of evaluating large amounts of data in the context of diagnosis, its use might increase quality and ensure objectivity of skin cancer diagnostics. AI algorithms can be trained with larger amounts of image data than even an experienced dermatologist can assess, which may increase the accuracy of the algorithm relative to physicians.

If used as an assistance tool, participants expected AI to be able to reduce error rates in diagnoses especially for less experienced physicians. They hoped that physicians would even be able to learn from the AI-based systems. In their opinion, the direct comparison might also motivate specialists to strive continuously to improve their own performance.

The use of AI-based tools might save time that physicians could use for more personal contact to the patient. Conversely, AI could allow first self-tests at home without visiting a physician—which could become even more important due to the increasing paucity of physicians. AI was also expected to improve processes, and to reduce the burden on healthcare by helping to avoid unnecessary diagnostics and treatments.

Participants also assumed that using AI in skin cancer diagnosis might ultimately result in more transparency, if AI-based tools were able to objectively quantify the likelihood of a lesion being malignant objectively in a way that patients could comprehend.

### Attitudes Toward Different Application Scenarios and Participation

**Figure 3:**
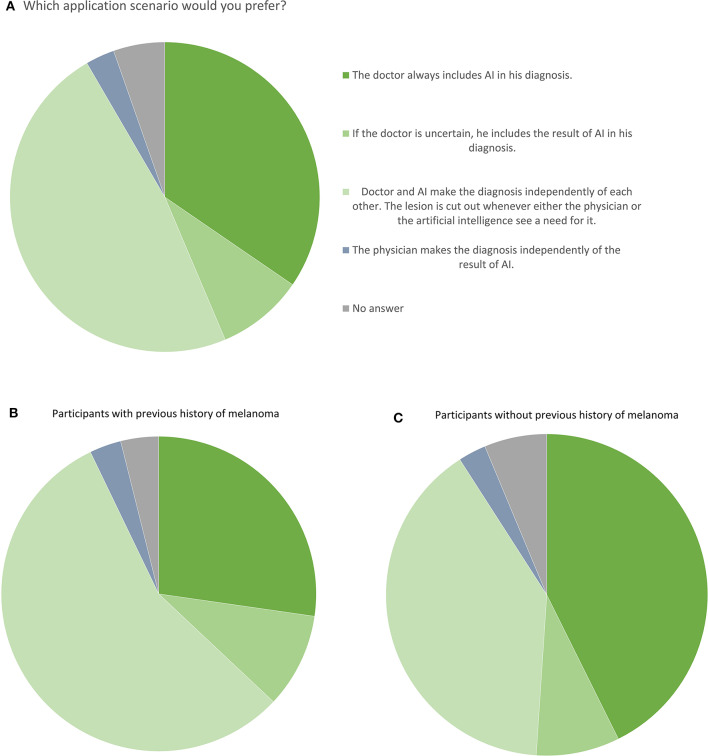
Attitudes toward different application scenarios. **(A)** Pie chart shows the percentages of all participants' preferences. **(B)** Pie chart shows the percentages of melanoma patients' preferences. **(C)** Pie chart shows the percentages of preferences of participants without previous history of melanoma.

Nearly half of all participants could envisage a diagnostic routine in which physicians and an AI-based assistance system classify the same lesion independently of each other and a tissue sample is taken when either of them rates the lesion as requiring further investigation. One third stated that they would like the physician to include the result of AI always in his or her diagnosis. Altogether, more than 90% of the participants endorsed the use of AI as supporting tool for the physician. Only a very small proportion, 3% of all participants, claimed that they would prefer not to incorporate AI in skin cancer diagnostics at all (Figure 3A).

**Figure 4:**
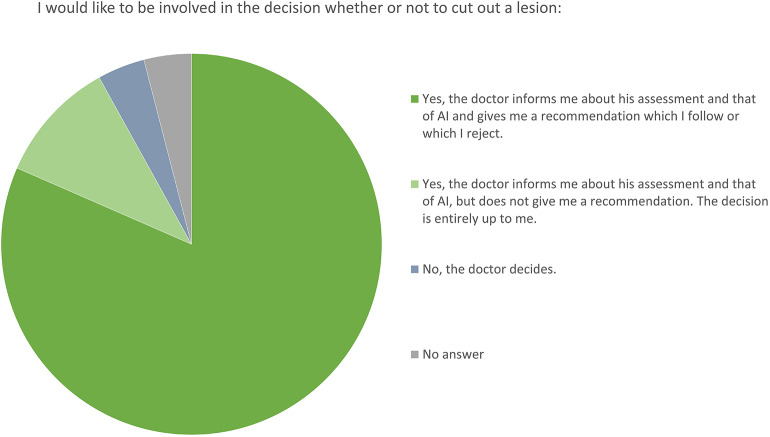
Attitudes toward patients' participation. Pie chart shows the percentages of participants' preferences.

When asked about their attitude toward shared decision-making, the vast majority of the patients (82%) stated that they would like to take their decision together with the physician on the basis of the results of the examination. About 10% would prefer to get all information and to take the decision on their own and only 4% of the participants would prefer to not be involved in the decision making at all (Figure 4).

### Associations Between Sociodemographic Data on Attitude and Awareness Toward AI: Subgroup Analyses

We prespecified sub-group analyses on age, education, gender, and participants with and without a history on melanoma. Subgroup analyses were conducted for each question for every subgroup. Overall, only minor differences were identified in the attitudes toward AI in medical use between the evaluated subgroups. Most of these differences appeared between participants with and without a history of melanoma.

There was no significant difference between age groups. Numerical differences between participants with higher and lower education levels were not statistically significant.

Sub-group analyses showed that more female than male participants would have the lesion removed if either the physician or the AI algorithm rated this as necessary (83–93% vs. 70–83%, *p* < 0.02).

Significantly more (about 97%) respondents with a previous history of melanoma would support the use of AI in medicine compared to non-melanoma patients (~91%) (*p* = 0.03). Moreover, they would be considerably more open to using AI applications for early detection even at home (around 66% compared to 46%, *p* = 0.004). Moreover, the majority of melanoma patients preferred an application scenario in which AI and the physician take the decision on a lesion independently from each other, whereas the majority of the non-melanoma patient respondents preferred the inclusion of AI results into the diagnosis by the physician (*p* = 0.027, see Figures 3B,C).

## Discussion

As the use of AI might lead to more precise, impartial and faster diagnostics, experts consider it likely that AI-driven technology will increasingly be implemented in the medical field in the near future, in particular in skin cancer diagnostics. A lack of acceptance by patients and physicians would have severely curtailed this development. In our survey amongst patients and putative patients regarding their attitudes and their awareness toward the use of AI in melanoma diagnostics, we found that the overwhelming majority of participants support the use of AI for medical applications. They expressed a high level of confidence in decision-making by AI. Importantly, although most participants in our survey thought that AI can contribute to improve diagnostic accuracy, they also believed that it cannot and should not replace the dermatologist. This ambivalence might reflect a general fear that AI might supplant the expertise and diagnostic skills of physicians and is concordant with the desire for personal contact with the treating physician, impersonal consultations were among the most common concerns mentioned in the survey. This may imply that AI-based applications as stand-alone systems in diagnosis would probably not be accepted. Along the same lines, the majority of German undergraduate medical students don't think that human radiologists will be replaced by AI (22).

While some participants also hope that the use of AI in diagnosis will lead to fewer unnecessary biopsies, many participants apparently see the biggest advantage of additional AI-based diagnostics in the detection of malignant lesions that might otherwise be overlooked by the physician performing the examination. This result is mainly triggered by the preference of the participants with previous history of melanoma to obtain separate assessment results from both the AI-based system and the physician about a pending biopsy. This might mean that additional diagnostic tools would primarily lead to more biopsies, a burden that the participants would accept for safety reasons. Although avoiding unnecessary biopsies is obviously also an important goal in melanoma early detection, it is important to take this into account when determining the optimal relationship between sensitivity and specificity and suitable cut-off values for an algorithm that might be applied in clinics. However, this result may not be representative of the situation in the general public, since our results also show that a previous cancer diagnosis may have altered patients' preferences in some respects. The answers of the participants could help to decide how such an algorithm should be implemented in the clinic, but they ought to be supplemented with the results of another survey targeting individuals with little previous contact with the skin cancer topic.

Either positioning the physician or the AI-based tool first, have advantages and disadvantages (23). But independent decisions could provide the benefits of both, increased sensitivity while maintaining current clinical workflows, provide an automated second opinion, reduce excessive dependence on AI, and take note of a possible discrepancy between AI and physician.

Somewhat surprisingly, more than half of the participants would be amenable to the use of AI at home, e.g., via a smartphone app. However, incorrect use, flawed or scientifically unsound applications or incomprehensible outputs and a lack of personal contact with the physician might increase rather than reduce stress and anxiety levels for users and the misclassifications of both benign and malignant lesions (24). Thus, AI-based diagnostic apps will have to be evaluated very carefully to determine their potential for benefits and harms. With people's desire for empowerment and autonomy through self-management in mind, it might be indispensable to develop digital end devices that enable independent, quality-assured and always available support without spatial and temporal barriers. If an AI-based skin diagnostics app did pass the required tests, patients might profit considerably from its use.

To encompass the requirement for transparent and comprehensible treatment decisions, it will be necessary to work on strategies that allow the results of AI to be interpreted and verified (at least in part). Due to the high complexity of the algorithms, a complete transparency of AI will probably not be possible, but it might be possible to explain the decisive influencing factors on individual decision steps within the algorithms (25, 26).

Altogether, most participants see the use of AI in the medical field in general and particularly in skin cancer diagnostics as a gain, especially for those areas that exceed human capabilities. However, they envisage it mainly as an assisting system that supports and complements a decision made by a human provider. Although the survey in this form may mainly reflect the view of people who have concerned themselves with the topic of skin cancer, the results may be valuable knowledge for the transfer of the diagnosis algorithms from research into clinical practice and further studies using AI in medical applications.

## Limitations

The sample size of this study (*n* = 298) is still relatively small. Also, more of the respondents were predominantly female, and more respondents had a high or very high educational level. Since half of the questionnaires were filled in by (former) melanoma patients and via self-help organizations, we cannot exclude the risk of sampling bias and the results that we obtained are probably not fully generalizable to the general population. Our approach was chosen to be able to gain a first impression of the difference between the attitudes given by melanoma patients or –survivors and those of participants without a history of melanoma. Since none of the survey questions was mandatory, not every participant answered every question. Some responders also did not reveal their sociodemographic data, so those data couldn't be included into the statistics in sub-group analyses.

## Conclusion

Most of the participants in our survey, both with and without a previous melanoma diagnosis, had a positive attitude toward the use of AI in the medical field in general and melanoma diagnostics in particular, especially when AI was applied as assistant system for the treating physician.

## Data Availability Statement

The datasets generated in this survey are available on request to the corresponding author.

## Ethics Statement

Ethics approval for the online survey was waived by the Ethics Committee of the University of Heidelberg due to the fact that all of the participants remained anonymous. Ethical approval for this study was not required in accordance with local legislation and national guidelines. The survey received approval by the Board of Data Protection at the German Cancer Research Center (DKFZ).

## Author Contributions

TJ, EK-H, AHe, and TB contributed to study concept and design. TJ carried out the implementation, data collection, analysis and statistics with contributions from EK-H and AHe. TH-L provided biostatistical advice. TJ and EK-H wrote the manuscript. AHe, MS, and RM contributed to design and distribution of the questionnaire. SF and TB supervised the project and obtained the funding. JU, AHa, DS, and WS and all other co-authors critically reviewed and edited the questionnaire and the manuscript.

## Conflict of Interest

TB would like to disclose that he is the owner of Smart Health Heidelberg GmbH (Handschuhsheimer Landstr. 9/1, 69120 Heidelberg, Germany) which develops mobile apps, outside of the submitted work. The remaining authors declare that the research was conducted in the absence of any commercial or financial relationships that could be construed as a potential conflict of interest.
