# Supplementary material for: Artificial Intelligence in Skin Cancer Diagnostics: The Patients' Perspective
Source: Front Med (Lausanne). 2020 Jun 2;7:233. doi: 10.3389/fmed.2020.00233 (PMC7326111; doi:10.3389/fmed.2020.00233)
Supplement: Supplementary file 1 [file Data_Sheet_1.PDF]

Hautdiagnostik durch Künstliche Intelligenz

In dieser Umfrage stellen wir Ihnen Fragen zu Ihrer Meinung, Befürchtung und Erwartung zum Thema **Künstliche Intelligenz bei der Erkennung von Hautkrankheiten**.

Sie benötigen dafür keinerlei Vorwissen zu Künstlicher Intelligenz.

Zum Ausfüllen des Fragebogens benötigen Sie etwa **10 Minuten**.

Sie füllen den Fragebogen **anonym** aus, somit ist keine Rückverfolgbarkeit auf Ihre Person möglich. Die Umfrage ist zudem **freiwillig**, durch die Nichtteilnahme entstehen Ihnen keinerlei Nachteile. Sie können auch Fragen unbeantwortet lassen.

Warum bitten wir Sie, an dieser Umfrage teilzunehmen?
Wir möchten in einem bundesweiten Forschungsprojekt neue Methoden entwickeln, um Hauterkrankungen und speziell Hautkrebs besser zu erkennen. Dazu möchten wir Künstliche Intelligenz einsetzen. Dabei ist es für uns wichtig zu wissen, welche Vorstellungen die Menschen in Deutschland von der Künstlichen Intelligenz haben und ob sie Künstliche Intelligenz überhaupt nutzen würden.

Wir freuen uns sehr, dass Sie uns Ihre Meinung über Künstliche Intelligenz mitteilen! Für weitere Fragen und Rückmeldungen stehen wir Ihnen gern zur Verfügung.
Kontakt: Dr. Tanja Jutzi (t.jutzi@dkfz.de)

Verantwortlich:
Dr. med. Titus Brinker
Deutsches Krebsforschungszentrum – DKFZ
Translationale Onkologie
Im Neuenheimer Feld 280
69120 Heidelberg

Hinweis: Die Umfragedaten werden innerhalb des DKFZ ausgewertet. Die Ergebnisse verbleiben bis zum Ende der Umfrage im DKFZ. Eine Gesamtauswertung wird veröffentlicht. In dieser Umfrage sind 16 Fragen enthalten.

Datenschutz

☐ Ich habe die Datenschutzhinweise von Limesurvey gelesen und bin einverstanden.

Angaben zur Person

Bitte geben Sie Ihr Geschlecht an.
Bitte wählen Sie eine der folgenden Antworten:

- Männlich
- Weiblich
- Divers
- Keine Antwort

Bitte geben Sie Ihr Alter an.
Bitte wählen Sie eine der folgenden Antworten:

- ≤ 30 Jahre
- 31 - 45 Jahre
- 46 - 60 Jahre
- 61 - 75 Jahre
- > 75 Jahre
- Keine Antwort

Ich bin Hautkrebs-Patient.
Bitte wählen Sie eine der folgenden Antworten:

- ja
- nein
- Keine Antwort

Was ist Ihr höchster Bildungsabschluss:
Bitte wählen Sie eine der folgenden Antworten:

- Ohne Abschluss
- Volks-/Hauptschulabschluss
- Mittlere Reife
- Abitur (bzw. Fachhochschulreife)
- Hochschulabschluss
- Keine Antwort

Ich habe eine Vorstellung, was Künstliche Intelligenz bedeutet.
Bitte wählen Sie eine der folgenden Antworten:

- Ich stimme ganz und gar zu
- Ich stimme teilweise zu
- Unentschieden
- Ich stimme eher nicht zu
- Ich stimme überhaupt nicht zu
- Keine Antwort

Künstliche Intelligenz zur Diagnose

Künstliche Intelligenz bedeutet, dass Computer auf Grundlage großer Datenmengen in der Lage sind, Probleme selbständig zu lösen. Sie wird unter anderem genutzt, um in Bildern automatisiert bestimmte Muster zu erkennen. Dabei können Systeme mit Künstlicher Intelligenz oft auch Unterscheidungsmerkmale aufdecken, die mit dem menschlichen Auge nur sehr schwer zu erkennen sind.

Antwortmöglichkeiten:
Ich stimme ganz und gar zu
Ich stimme teilweise zu
Unentschieden
Ich stimme eher nicht zu
Ich stimme überhaupt nicht zu
Keine Antwort

Was wissen Sie zu Künstlicher Intelligenz und welche Meinung haben Sie dazu?

- Ich habe schon von Beispielen zum Einsatz der Künstlichen Intelligenz in der Medizin gehört.

- Ich befürworte den Einsatz von Künstlicher Intelligenz in der Medizin, wenn ich als Patient einen Nutzen durch bessere und frühere Diagnosen habe.

- Ich befürworte den Einsatz von Künstlicher Intelligenz in der Medizin, wenn ich als Patient einen Nutzen durch bessere Heilungschancen habe.

Wenn Künstliche Intelligenz zwischen Bildern von schwarzem Hautkrebs und einem harmlosen Muttermal sehr gut unterscheiden kann, würde ich die Künstliche Intelligenz zur Früherkennung von Hautkrebs nutzen.

- Beim Arzt als unterstützendes System
- Beim Arzt als alleiniges System
- Selbst zu Hause, z.B. per Smartphone App

- Wenn die Krankenkasse die Kosten dafür nur teilweise übernimmt, wäre ich bereit, dafür Zuzahlungen zu leisten.

- Ich würde eigene Gesundheitsdaten anonymisiert für die Forschung zur Verfügung stellen, damit die Künstliche Intelligenz bessere Diagnosen für andere Patienten erstellen kann.

Aufgrund der schwierigen Unterscheidung zwischen harmlosen Muttermalen und dem schwarzen Hautkrebs werden Muttermale oftmals zu häufig herausgeschnitten. Andererseits kommt es in seltenen Fällen auch vor, dass schwarzer Hautkrebs zu spät erkannt wird.
In Forschungsexperimenten wurde gezeigt, dass Künstliche Intelligenz sehr zuverlässig zwischen Hautkrebs und harmlosen Muttermalen unterscheiden kann.
Vertrauen Sie auf Künstliche Intelligenz?

- Künstliche Intelligenz sollte zur Unterstützung vom Arzt eingesetzt werden, um die Diagnose noch sicherer zu machen.

Angenommen, die Künstliche Intelligenz kann **genau so gut** zwischen Hautkrebs und harmlosen Muttermalen unterscheiden wie die Ärzte:

- Der Arzt sieht keinen Verdacht auf Hautkrebs, die Künstliche Intelligenz dagegen schon. Ich würde das Muttermal für weitere Untersuchungen herausschneiden lassen.

- Die Künstliche Intelligenz sieht keinen Verdacht auf Hautkrebs, der Arzt dagegen schon. Ich würde das Muttermal für weitere Untersuchungen herausschneiden lassen.

Angenommen, die Künstliche Intelligenz kann **besser** zwischen Hautkrebs und harmlosen Muttermalen unterscheiden als die Ärzte:

- Der Arzt sieht keinen Verdacht auf Hautkrebs, die Künstliche Intelligenz dagegen schon. Ich würde das Muttermal für weitere Untersuchungen herausschneiden lassen.
- Die Künstliche Intelligenz sieht keinen Verdacht auf Hautkrebs, der Arzt dagegen schon. Ich würde das Muttermal für weitere Untersuchungen herausschneiden lassen.

Freitextfelder:

Welche Bedenken hätten Sie bei der Nutzung der Künstlichen Intelligenz in der Medizin?

Welche Erwartungen setzten Sie in die Nutzung der Künstlichen Intelligenz in der Medizin?

Mögliche Anwendungsbeispiele

Stellen Sie sich die folgende Untersuchung vor. Bei der Haut-Untersuchung von Muttermalen entscheidet der Arzt, ob ein Verdacht auf Hautkrebs besteht und daher das Muttermal für weitere Untersuchungen herausgeschnitten werden sollte. Zusätzlich entscheidet die Künstliche Intelligenz, ob das Muttermal herausgeschnitten werden sollte.

Welche Vorgehensweise würden Sie wählen, vorausgesetzt, die Künstliche Intelligenz unterscheidet sehr genau zwischen Hautkrebs und harmlosen Muttermalen:

Bitte wählen Sie eine der folgenden Antworten:

- [ ] Der Arzt trifft die Diagnose, unabhängig vom Ergebnis der Künstlichen Intelligenz.

- [ ] Wenn der Arzt unsicher ist, bezieht er das Ergebnis der Künstlichen Intelligenz in seine Diagnose mit ein.

- [ ] Der Arzt bezieht die Künstliche Intelligenz immer in seine Diagnose mit ein.

- [ ] Arzt und Künstliche Intelligenz stellen die Diagnose unabhängig voneinander. Herausgeschnitten wird das Muttermal immer dann, wenn entweder der Arzt oder die Künstliche Intelligenz eine Notwendigkeit dazu sehen.

- [x] Keine Antwort

Bei der Entscheidung, ob ein Muttermal herausgeschnitten wird oder nicht, möchte ich beteiligt sein.

Bitte wählen Sie eine der folgenden Antworten:

- [ ] Nein, der Arzt entscheidet.

- [ ] Ja, der Arzt klärt mich über seine Einschätzung und die der Künstlichen Intelligenz auf und gibt mir eine Empfehlung, der ich folge oder die ich ablehne.

- [ ] Ja, der Arzt klärt mich über seine Einschätzung und die der Künstlichen Intelligenz auf, gibt mir aber keine Empfehlung. Die Entscheidung liegt vollständig bei mir.

- [x] Keine Antwort

-Vielen Dank für Ihre Teilnahme-
